# Regulatory Proteolysis in *Arabidopsis*-Pathogen Interactions

**DOI:** 10.3390/ijms161023177

**Published:** 2015-09-24

**Authors:** Miklós Pogány, Tamás Dankó, Evelin Kámán-Tóth, Ildikó Schwarczinger, Zoltán Bozsó

**Affiliations:** Plant Protection Institute, Centre for Agricultural Research, Hungarian Academy of Sciences, Herman Ottó út 15, 1022 Budapest, Hungary; E-Mails: danko.tamas@agrar.mta.hu (T.D.); toth.evelin@agrar.mta.hu (E.K.-T.); schwarczinger.ildiko@agrar.mta.hu (I.S.); bozso.zoltan@agrar.mta.hu (Z.B.)

**Keywords:** *Arabidopsis*, pathogen, protease, effector, immunity, cell death, *Pseudomonas syringae*

## Abstract

Approximately two and a half percent of protein coding genes in *Arabidopsis* encode enzymes with known or putative proteolytic activity. Proteases possess not only common housekeeping functions by recycling nonfunctional proteins. By irreversibly cleaving other proteins, they regulate crucial developmental processes and control responses to environmental changes. Regulatory proteolysis is also indispensable in interactions between plants and their microbial pathogens. Proteolytic cleavage is simultaneously used both by plant cells, to recognize and inactivate invading pathogens, and by microbes, to overcome the immune system of the plant and successfully colonize host cells. In this review, we present available results on the group of proteases in the model plant *Arabidopsis thaliana* whose functions in microbial pathogenesis were confirmed. Pathogen-derived proteolytic factors are also discussed when they are involved in the cleavage of host metabolites. Considering the wealth of review papers available in the field of the ubiquitin-26S proteasome system results on the ubiquitin cascade are not presented. *Arabidopsis* and its pathogens are conferred with abundant sets of proteases. This review compiles a list of those that are apparently involved in an interaction between the plant and its pathogens, also presenting their molecular partners when available.

## 1. Introduction

Proteolytic enzymes have been proven to possess crucial housekeeping and regulatory functions in cells of intact or pathogen-exposed plant tissues. Enzyme-catalysed proteolysis in living cells, however, is performed by an extremely diverse group of enzymes. In the genome of the dicotyledonous model plant *Arabidopsis thaliana*, the number of protease coding genes and their homologs is between 800 and 900, representing approximately 2.5% of all protein-coding genes [1,2,3]. In humans, proteases also represent about 2% of protein coding genes [4].

Proteases (peptidases or proteolytic enzymes) cleave peptide bonds between amino acid residues of proteins, oligo- or polypeptides. Aminopeptidases detach N-terminal amino acid residues, carboxypeptidases split C-terminal amino acid residues and endopeptidases cleave peptide bonds between amino acids in internal positions. Proteases in *Arabidopsis* hydrolyze peptide bonds in five ways, which gives the names to five catalytic classes: aspartic proteases, cysteine proteases, serine proteases, metalloproteases and threonine proteases [1,3,5].

A systematic classification of peptidases is offered by the MEROPS database (release 9.13) [1] including 745 known and putative peptidases and their 124 homologs for *Arabidopsis thaliana*. These *Arabidopsis* proteases are distributed over 30 clans and subdivided into 60 families [1,2].

The aim of this article, on one hand, is to provide an organised overview on the proteolytic enzymes produced by *Arabidopsis* cells whose functions in interactions with pathogens have been confirmed. Pathogen-derived proteolytic factors are also discussed when they apparently play regulatory roles during interactions with their host. The nature of interaction is typically proteolytic cleavage of host metabolites. However, besides their catalytic domains, a great number of proteases contain numerous additional domains or modules [4], which enable them to establish a wide range of interactions.

Host-derived *Arabidopsis* proteolytic enzymes discussed in the article are listed in Table 1. Proteolytic enzymes secreted by pathogens of *Arabidopsis* are summarized in Table 2.

It has to be emphasized that reviewing functional aspects of the highly sophisticated ubiquitin-26S proteasome system (UPS) in *Arabidopsis* pathogenesis was beyond the scope of this article. Although UPS is an extremely important cellular proteolytic machinery, and our knowledge on the involvement of the UPS in plant-pathogen interactions has exploded in the last 10 years, there have been several excellent review papers written in this particular field recently [6,7,8,9,10]. Our primary goal with the current article was to compile published data on proteins with experimentally proven or computationally inferred peptidase activity in the context of *Arabidopsis* microbial pathogenesis. The vast majority of UPS components do not fall into this category. However, the *Arabidopsis* proteasome β1 subunit PBA1 is still discussed here because it apparently possesses caspase-3-like proteolytic activity [11].

Some clearly-defined types and aspects of *Arabidopsis*-pathogen interactions are discussed throughout the paper. These are (i) pattern-triggered or basal immunity, when conserved microbe-associated molecular patterns (MAMPs) are recognized by host cell surface pattern-recognition receptors (PRR); (ii) susceptibility, when virulent or compatible pathogens deliver effectors or virulence factors into the host cells overcoming host basal immunity and thereby causing disease; (iii) effector-triggered or hypersensitive response (HR)-type immunity, when effectors released by an avirulent or incompatible pathogen strain are detected in the host cells by intracellular nucleotide-binding leucine-rich repeat (NB-LRR) immune receptor R proteins leading to programmed cell death (PCD) or HR and (iv) systemic acquired resistance (SAR), when an initial pathogen infection is able to induce a primed condition in local (infected) and distal tissues of host plants to make them respond in a more rapid and robust manner to even lower levels of pathogenic cues than control plants do [12,13].

**Table 1:** *Arabidopsis thaliana* proteases whose functions in interactions with pathogens have been confirmed.

Gene	Full Name	AGI Code	Uniprot Accession	MEROPS Identifier	Reference
*AtCDR1*	*Constitutive Disease Resistance 1*	*At5g33340*	Q6XBF8	A01.069	[14,15]
*AtAED1*	*Apoplastic EDS1-Dependent 1*	*At5g10760*	Q9LEW3	A01.A14	[16]
*AtRD21a*	*Responsive to Dehydration 21a*	*At1g47128*	P43297	C01.064	[17,18,19,20]
*AtRD21b*	*Responsive to Dehydration 21b*	*At5g43060*	Q0WM94	C01.A12	[20]
*AtXCP1*	*Xylem Cysteine Proteinase 1*	*At4g35350*	O65493	C01.065	[17]
*AtXCP2*	*Xylem Cysteine Proteinase 2*	*At1g20850*	Q9LM66	C01.120	[17,20]
*AtCPR1*	*Probable Cysteine Proteinase*	*At3g19400*	Q9LT77	C01.A12	[17]
*AtALEU*	*Aleurain*	*At5g60360*	Q8H166	C01.163	[17]
*AtALEUL*	*Aleurain-Like*	*At3g45310*	Q8RWQ9	C01.162	
*AtRD19a*	*Responsive to Dehydration 19a*	*At4g39090*	P43296	C01.022	[21]
*AtCathB1*	*Cathepsin B1, B2, B3*	*At1g02300*	Q56XY7	C01.A10	[22]
*AtCathB2*		*At1g02305*	Q93VC9	C01.144	
*AtCathB3*		*At4g01610*	Q9ZSI0	C01.144	
*AtMC1*	*Metacaspase 1*	*At1g02170*	Q7XJE6	C14.047	[23]
*AtMC2*	*Metacaspase 2*	*At4g25110*	Q7XJE5	C14.A04	[23]
*AtMC4*	*Metacaspase 4*	*At1g79340*	O64517	C14.033	[24]
*AtαVPE*	*α, β, δ, or γ Vacuolar Processing Enzyme*	*At2g25940*	P49047	C13.002	[25,26,27,28]
*AtβVPE*		*At1g62710*	Q39044	C13.001	
*AtδVPE*		*At3g20210*	Q9LJX8	C13.A01	
*AtγVPE*		*At4g32940*	Q39119	C13.006	
*AtCEP1*	*KDEL Cysteine Endopeptidase 1*	*At5g50260*	Q9FGR9	C01.A03	[29]
*AtSBT3.3*	*Subtilase 3.3*	*At1g32960*	Q9MAP5	S08.A35	[30]
*AtPBA1*	*26S Proteasome β1 Subunit*	*At4g31300*	Q8LD27	T01.010	[31]

**Table 2:** Pathogen-secreted proteases whose functions in interactions with *Arabidopsis* have been partially elucidated.

Protease	Species	Uniprot Accession	MEROPS Identifier	Reference
AvrPphB	*P. syringae* pv. *phaseolicola*	Q52430	C58.002	[32,33,34]
AvrRpt2	*P. syringae* pv. *tomato*	Q6LAD6	C70.001	[35,36,37,38,39,40,41]
XopD	*X. campestris* pv. *vesicatoria*	Q3BYJ5	C48.023	[42,43]
HopX1	*P. syringae* pv. *tabaci*	Q83YM6	N/A	[44]
protease IV	*P. aeruginosa*	Q02SZ7	S01.281	[45]
AprA	*P. syringae* pv. *tomato*	Q87ZU2	M10.060	[46]

## 2. Functions of Proteolytic Enzymes in Various *Arabidopsis thaliana* Pathosystems

### 2.1. Host-Derived Aspartic Proteases

The majority of proteases discussed in this review are cysteine proteases. Nevertheless, one of the first *Arabidopsis* proteases proven to function in the immune system was an exception. Overexpression of the aspartic protease **C**onstitutive **D**isease **R**esistance **1** (CDR1) in a gain-of-function mutant resulted in resistance to normally virulent *Pseudomonas syringae*. Antisense *CDR1* plants with reduced levels of CDR1 protein were compromised for resistance to avirulent *P. syringae* and more susceptible to virulent strains than wild type. Levels of salicylic acid were significantly elevated in *CDR1* overexpressor compared with wild-type plants and CDR1 activation apparently induced a salicylic acid-dependent disease resistance response in *Arabidopsis* [14,15]. CDR1 encodes an apoplastic protein that shares significant sequence similarity to aspartic proteases. It was also shown that the proteolytic activity of CDR1 was necessary for its biological functions. Like other eukaryotic aspartic proteases, CDR1 possesses two active sites with the conserved motifs aspartic acid-threonine-glycine-serine and aspartic acid-serine-glycine-threonine, respectively. These data indicate that CDR1 encodes an aspartic protease that functions biologically by the proteolytic cleavage of its endogenous target. CDR1 might process a cell surface protein that could be a component of the basal host defense complex or alternatively it may release an extracellular mobile peptide elicitor that activates host basal defense responses [14,15]. Although the natural substrates for CDR1 are still missing, recombinant CDR1 activity is considerably increased by redox-dependent, disulfide-mediated dimerization [47].

Another aspartic protease, **A**poplastic **E**DS1-**D**ependent **1** (AED1) was recently described [16], searching for systemic acquired resistance (SAR) regulatory proteins and using two-dimensional PAGE. AED1 accumulation together with some other proteins was reduced in the *eds1* mutant expressing the *P. syringae* effector AvrRpm1 in comparison with wild type background *AvrRpm1* expressing plants. Although the mode of AED1 action (e.g., its cellular targets) is still missing, it was concluded that it functions as a negative regulator of systemic acquired resistance acting downstream of salicylic acid. By cleaving apoplastic proteinaceous substrates, it might be part of a homeostatic mechanism to limit SAR signaling and thus regulating the resource allocation in the tradeoff between defense and plant growth [16].

### 2.2. Host-Derived Cysteine Proteases

Cysteine proteases contain a cysteine nucleophilic residue in their active site that performs a nucleophilic attack in the first step of proteolysis resulting in an intermediate state where the enzyme is covalently attached to its substrate [48]. Known and putative cysteine protease sequences compose almost 16% of all listed peptidase sequences in the latest release of the MEROPS database [1]. Twenty-three out of the 29 proteases discussed in this current review also belong to the group of cysteine proteases. Protease activity profiling [48,49,50] was applied to investigate whether the fungal effector Avr2 produced by *Cladosporium fulvum* is able to inhibit *Arabidopsis* cysteine proteases [17]. Protein extracts prepared from *Arabidopsis* plants were treated with DCG-04, a biotinylated derivative of the irreversible cysteine protease inhibitor E-64. DCG-04 reacts with the catalytic cysteine residue of cysteine proteases and locks the cleavage mechanism in the covalent intermediate state. The biotinylated cysteine proteases were subsequently detected on protein gel blots using a conjugate of streptavidin with horseradish peroxidase. Avr2 was previously shown to bind and inhibit the tomato cysteine protease Rcr3 [51]. Indeed, Avr2 exhibited marked inhibitory effect on most detected *Arabidopsis* cysteine proteases except Cathepsin B3. Xylem Cysteine Proteinase 1 (XCP1), Xylem Cysteine Proteinase 2 (XCP2) and a Probable Cysteine Proteinase CPR1 showed high Avr2 affinity, whereas **R**esponsive to **D**ehydration 21A (RD21A) and thiol proteases Aleurain and Aleurain-Like had lower but still apparent affinity to Avr2 [17]. Lack of RD21A cysteine protease activity in *Arabidopsis* T-DNA mutants led to increased susceptibility to the necrotrophic fungus *Botrytis cinerea* [18]. RD21A was also shown to be a partner of cytochrome c during hydrogen peroxide-induced programmed cell death in cultured *Arabidopsis* cells [19]. XCP2, RD21A and **R**esponsive to **D**ehydration 21B (RD21B) were also independently identified by yeast two-hybrid assays as interacting partners of the *Arabidopsis* PIRIN2 protease inhibitor encoded by locus *At2g43120* [20]. Functional aspects of the XCP2-PIRIN2 interaction were further investigated revealing that PIRIN2 inhibits the autolytic degradation of XCP2. This stabilization of XCP2 by PIRIN2 results in accumulation of XCP2 and in increased overall XCP2 activity. It was also presented that the XCP2-PIRIN2 interaction is necessary for full susceptibility to the xylem-colonizing bacterial pathogen *Ralstonia solanacearum*. It is conceivable, that XCP2-mediated autolysis of cellular contents in leaves or vessel elements facilitates *R. solanacearum* pathogenesis [20].

The cysteine-type endopeptidase **R**esponsive to **D**ehydration 19A (RD19A) was identified as an interacting partner of *R. solanacearum* PopP2 effector [21]. RD19A encodes a drought-inducible cysteine protease [52] whose transcript levels increase strongly after *R. solanacearum* infection [21]. PopP2 elicits a disease resistance response in *Arabidopsis* mediated by its cognate R protein RRS1-R and functional RD19A is required for efficient RRS1-R-dependent defense against *R. solanacearum*. As far as the intracellular position of RD19A, without PopP2 it localizes in mobile prevacuolar vesicles, showing perfect colocalization with another cysteine protease Aleurain. In the presence of PopP2 effector, however, RD19A is recruited to the nucleus where it physically associates with PopP2 as it was confirmed by FLIM (Fluorescence Lifetime Imaging) approach [53]. The FLIM approach is a quantitative, noninvasive method that monitors the Förster resonance energy transfer between the donor and acceptor molecules fused to PopP2 and RD19A, respectively. Collectively, these findings suggest that RD19A is an important *Arabidopsis* factor for PopP2-triggered RRS1-R–mediated disease resistance.

Cathepsins are lysosomal cysteine and aspartic proteases that (along with caspases) participate in mammalian apoptosis [54]. Functions of three *Arabidopsis* orthologs of mammalian *Cathepsin B* (*CathB*) were analyzed to describe their contributions to various forms of disease resistance [22]. *AtCathB1*, *AtCathB2* and *AtCathB3* encode Cathepsin B-like cysteine proteases. Basal immunity to the virulent bacterial strain *P. syringae* pv. *tomato* DC3000 was not affected in *atcathb* single or double mutants, where only one or two *AtCathB* isoforms were knocked out. Triple mutants, on the other hand, exhibited impaired resistance to the virulent *P. syringae* strain, indicating that the three *AtCathB* isoforms in *Arabidopsis* act redundantly (they fulfill nearly the same role) to confer basal defense to the plants. (Triple mutants were generated by crossing *atcathb1* and *atcathb3* single mutants and the resulting double mutant was transformed with a CathB2:RNAi hairpin silencing construct.) Using an incompatible *Arabidopsis*-*P. syringae* pv. *tomato* system (*RPM1*-*AvrB*), it was also presented that *AtCathB* genes do not contribute to *R* gene mediated resistance, but they are redundantly required for programmed cell death (PCD) during the hypersensitive response (HR) triggered by *P. syringae* pv. *tomato* DC3000 (*AvrB*) [22].

Metacaspases are distant orthologs of animal caspases in plants, which are also present in protozoa and fungi. They belong to the CD cysteine protease clan [1]. Nine metacaspase genes (*AtMC1*–*AtMC9*) have been found in the *Arabidopsis* genome [3]. The role of metacaspases in pathogen-induced (as well as in other forms of) programmed cell death in *Arabidopsis* is now well established. AtMC1 is a crucial pro-death protein in *Arabidopsis* during the hypersensitive response mediated by intracellular NB-LRR immune receptor R proteins. T-DNA insertional mutation in *AtMC1* resulted in markedly reduced cell death upon elicitation by the avirulent bacterial strain *P. syringae* pv. *tomato* DC3000 (*avrRPM1*). In contrast, AtMC2 negatively regulated the same cell death phenomenon and it antagonized AtMC1 function. AtMC1 activity required conserved caspase-like catalytic residues, whereas AtMC2 function appeared to be independent of the putative catalytic residues [23].

AtMC4 was also confirmed as a positive mediator of cell death in *Arabidopsis* tissues challenged by avirulent bacteria or treated with a fungal toxin [24]. Plants carrying mutation in *atmc4* showed not only decreased sensitivity to the mycotoxin fumonisin B1 produced by the fungal pathogen *Fusarium moniliforme*, but they also developed reduced hypersensitive cell death symptoms when they were inoculated with the avirulent bacterial strain *P. syringae* pv. *maculicola* (*avrRpt2*). Subcellular localization studies revealed that mature AtMC4 resides mainly in the cytoplasm [24]. Another *Arabidopsis* metacaspase, AtMC9 was currently described as a proteolytic enzyme responsible for cleavage and activation of the GRIM REAPER protein that controls superoxide-induced cell death in *Arabidopsis*. Both GRIM REAPER and AtMC9 show extracellular localization [55].

Vacuolar processing enzyme (VPE) is a cysteine proteinase originally identified as the proteinase responsible for the maturation and activation of vacuolar proteins in plants [56]. Unlike metacaspases, that lack aspartic acid specificity of caspases and cleave their substrates after arginine and lysine residues, VPEs cleave peptide bonds at the C-terminal sides of asparagine or aspartic acid residues [49]. The *Arabidopsis* genome encodes four *VPE* genes: *αVPE*, *βVPE*, *γVPE*, and *δVPE* [25]. A *VPE* quadruple mutant (deficient in transcribing functional mRNA for all four *VPE* isoforms simultaneously) showed greatly diminished fumonisin B1-induced cell death and analysis of single mutants revealed that *γVPE* possessed the most essential role in the fungal toxin-induced cell death [25]. In accord with these results, plants overexpressing *γVPE* exhibited increased ion leakage (a marker of hypersensitive cell death) after inoculation with the avirulent *P. syringae* pv. *tomato* DC3000 (*AvrRpm1*). This result suggests that *γVPE* regulates cell death progression during plant-pathogen interaction. Compromised resistance to *Botrytis cinerea*, *P. syringae* pv. *tomato* DC3000 (*AvrRpm1*) and turnip mosaic virus observed in a *γVPE* single mutant was also reported here [26].

Using VPE activity profiling, enhanced VPE activity in *Arabidopsis* plants challenged by a virulent strain (Noco2) of the oomycete pathogen *Hyaloperonospora arabidopsidis* was reported [27]. By contrast, an avirulent isolate (Cala2) of the same pathogen was unable to trigger VPE activation. Sporulation of *H. arabidopsidis* (virulent strain) was quantified in a *VPE* quadruple mutant and significant reduction in the spore count was observed in comparison with wild type plants, demonstrating that VPEs are needed to promote *H. arabidopsidis* virulence. These data indicate that *H. arabidopsidis* as an obligate biotroph may take advantage of increased protein turnover and nutrient release mediated by host VPE activation [27]. Interestingly, VPEs also promote mutualistic interaction with the fungus *Piriformospora indica* in *Arabidopsis* roots [28].

The involvement of an *Arabidopsis* KDEL cysteine endopeptidase (AtCEP1) in pathogen defense has been recently published [29]. KDEL cysteine peptidases are ubiquitous in plants and are characterized by a C-terminal lysine-aspartic acid-glutamic acid-leucine (KDEL) motif that serves as an endoplasmic reticulum retention signal [57]. AtCEP1 apparently accumulated in the endoplasmic reticulum of epidermal cells that were penetrated by the biotrophic powdery mildew fungus *Erysiphe cruciferarum*. AtCEP1 labeling was particularly strong around established fungal haustoria. Microscopic examination of plant-fungal interaction sites in an *atcep1* mutant revealed that epidermal cell death was suppressed, whereas the number of haustoria increased in infected leaves of the mutant genotype compared to wild type leaves. Taken together, these findings suggest roles for AtCEP1 in the appearance of late stages of host cell death during *E. cruciferarum* pathogenesis and potentially in plant basal resistance to restrict parasitic growth of a compatible powdery mildew [29].

### 2.3. Host-Derived Serine Protease

Subtilases (or subtilisin-like serine proteases) are a large family of serine proteases universal to all kingdoms of life but found most extensively in plants compared to other organisms [58]. The family type peptidase is subtilisin that was originally purified from strains of *Bacillus subtilis* and related bacteria [1,59]. The *Arabidopsis* genome contains 56 subtilase encoding loci, divided into six subfamilies [58]. A recently published set of results have underscored the importance of an extracellular *Arabidopsis* subtilase SBT3.3 in induced disease resistance [30]. Independent *SBT3.3* mutants with impaired SBT3.3 activity showed enhanced susceptibility to *P. syringae* pv. *tomato* DC3000 and to *H. arabidopsidis* (isolate WACO9). Conversely, overexpression of *SBT3.3* conferred enhanced resistance to bacterial and oomycete pathogens. When the *SBT3.3* overexpression phenotype was examined in a *sid2* or *npr1* mutant background (affecting salicylic acid biosynthesis or signaling), the enhanced disease resistance was abrogated, indicating that SBT3.3 operates upstream of the salicylic acid pathway. *SBT3.3* overexpression also poised salicylic acid-mediated defense genes for enhanced activation upon inoculation with *P. syringae* pv. *tomato* DC3000. It has been concluded, that SBT3.3 functions as a major component and positive regulator of salicylic acid-dependent immune priming, keeping cells in a sustained sensitized mode following a prior pathogen attack [30]. Intriguingly, tobacco and rice subtilisin-like serine proteases (named phytaspases) that are phylogenetically related to subtilases listed in *Arabidopsis* subtilase subfamily 1 were shown to regulate programmed cell death during abiotic stress or virus infection. Phytaspase is secreted from the cell and stored in the apoplast but is uniquely relocalized into the cytoplasm upon induction of programmed cell death, where it contributes to the cellular suicide machinery, presumably by cleaving its intracellular targets [60,61].

### 2.4. Arabidopsis PBA1, the β1 Subunit of the 26S Proteasome

The β1 subunit of the 26S proteasome in *Arabidopsis* is encoded by the *PBA1* gene (*At4g31300*) [62]. When *Arabidopsis* leaves were inoculated with avirulent phytopathogenic bacterial strains, leaf cells developed the fusion of membranes of their large central vacuoles with the plasma membranes, leading to the discharge of vacuolar proteins to the intercellular space [31]. This discharge of vacuolar fluid prevented proliferation of bacterial pathogens and activated programmed cell death. The described cellular response could be precisely connected to the activity of the β1 catalytic subunit (PBA1) of the 26S proteasome system, which system selectively breaks down proteins targeted for degradation by modification with polymers of ubiquitin through an ubiquitin-activating (E1) to ubiquitin-conjugating (E2) to ubiquitin ligase (E3) enzymatic cascade. The 26S proteasome is formed by two distinct particles: the 20S core proteasome and the 19S regulatory particle. The 20S core proteasome possesses two peripheral and two central rings and the two central rings are composed of seven β subunits, including β1, β2 and β5. These three subunits are responsible for the three proteolytic activities of the 26S proteasome (peptidylglutamyl peptide-hydrolyzing, trypsin-like, and chymotrypsin-like activities) that can selectively digest target proteins to short peptides [9,62,63]. The *Arabidopsis* 26S β1 subunit (PBA1) exhibits caspase-3-like or DEVDase proteolytic activity [31], recognizing aspartic acid-glutamic acid-valine-aspartic acid (DEVD) tetrapeptide sequences and hydrolyzing peptide bonds on the carboxy side of the second aspartic acid residue. This protease belongs to the threonine protease T1 (proteasome) family [1]. Depletion of PBA1 in three independent RNAi lines was able to suppress membrane fusion, vacuole discharge and hypersensitive cell death in tissues challenged by the avirulent bacterial strain *P. syringae* pv. *tomato* DC3000 (AvrRpm1). Monitoring growth of bacterial cells in the RNAi lines revealed that reduced PBA1 activity resulted in increased bacterial growth. Inoculation with the virulent *P. syringae* pv. *tomato* DC3000, on the contrary, did not cause different ultrastructural responses or markedly altered pathogen growth between wild type and *pba1* RNAi plants, suggesting that PBA1 activity is mostly required for *R* gene-mediated immunity and cell death in *Arabidopsis*. Results with a second avirulent *Arabidopsis*-*P. syringae* pv. *tomato* DC3000 (avrRpt2) interaction also corroborated conclusions described above [31].

These findings indicate that the PBA1 proteasome subunit acts as a caspase-3-like enzyme in *Arabidopsis*, regulating membrane fusion of the vacuolar and plasma membranes. This PBA1-mediated cellular response leads to hypersensitive cell death and resistance to avirulent bacterial pathogens [31]. Caspase-3 is involved in the execution of apoptosis in animal cells [64].

### 2.5. Cysteine Protease Effectors Secreted by Pathogens of Arabidopsis

AvrPphB (earlier designated as AvrPph3) is a bacterial type III effector, originally identified from *P. syringae* pv. *phaseolicola*. It triggers a disease resistance response and hypersensitive cell death in *Arabidopsis* plants expressing a corresponding immune receptor R protein RPS5 [65]. AvrPphB is a cysteine protease that cleaves the *Arabidopsis* protein kinase PBS1. Proteolytic cleavage of PBS1 by AvrPphB elicits an RPS5-mediated immune response and HR-type cell death. It has been shown by using a coimmunoprecipitation assay, that AvrPphB and PBS1 physically interact [32]. PBS1 cleavage was suggested to change the ATP *versus* ADP binding functions of RPS5 serving as a molecular switch to activate R-gene-mediated molecular pathway [33]. In accord with the guard model of R protein activation, RPS5 does not recognize the *Pseudomonas syringae* effector AvrPphB directly, but rather perceives the conformational modification of its cellular target (PBS1) as a result of AvrPphB proteolytic activity. It has been also discovered later that besides PBS1, there are other PBS1-like cellular targets of AvrPphB in *Arabidopsis*, such as BIK1, PBL1 or PBL2 receptor-like protein kinases [34]. These are also proteolytically cleaved by AvrPphB for inhibition of host pattern-triggered immunity.

AvrRpt2, another *P. syringae* type III effector with cysteine protease activity triggers a disease resistance response in *Arabidopsis* plants carrying the R protein RPS2 [35,36]. AvrRpt2 cleaves the *Arabidopsis* RIN4 protein, which leads to its elimination monitored by RPS2 [37,38,39]. Interestingly, RIN4 is attacked by several *P. syringae* effectors (AvrB, AvrRpm1, AvrRpt2, AvrPto, AvrPtoB, HopF2), highlighting its key role as a regulator of plant immunity [40,66]. Degradation of RIN4 by AvrRpt2 may prevent detection of RIN4 modification caused by other effectors, such as AvrB or AvrRpm1 in the presence of another immune receptor R protein RPM1 [39]. It was recently shown, that AvrRpt2 also promotes an auxin response in *Arabidopsis* by stimulating the degradation of auxin transcription repressor proteins (e.g., AXR2 or AXR3), and this function requires the cysteine protease activity of AvrRpt2 [41]. This AvrRpt2-dependent turnover of auxin transcription repressor proteins supports virulence of the compatible *P. syringae* pv. *tomato* DC3000 strain in susceptible *Arabidopsis* host.

An *Xanthomonas campestris* pv. *vesicatoria* type III effector, XopD exhibits small ubiquitin-like modifier (SUMO) protease activity, due to a cysteine protease domain located at its C-terminus [67]. XopD is a potent suppressor of *Arabidopsis* defense responses by accumulating in subnuclear structures called nuclear bodies in the nucleus of host cells and recruiting and binding *Arabidopsis* transcription factor MYB30 there, establishing a physical interaction with it [42]. MYB30 is a positive regulator of pathogen defense and hypersensitive response-related genes [68,69,70]. XopD was shown to repress the transcriptional activity of MYB30 and the defense of *Arabidopsis* plants exhibited against *Xanthomonas campestris*. These functions were, however, independent of the effector’s cysteine protease domain but dependent on an XopD helix–loop–helix (HLH) domain, suggesting that the cysteine protease domain might be involved in targeting host defense-related factors other than MYB30 [42].

In accord with this suggested model, a recent work presented *Arabidopsis* HFR1, a basic helix–loop–helix transcription factor as a potential host target regulated specifically by SUMO protease activity of XopD [43]. A truncated version of XopD (XopD*_Xcc_*_8004_), which lacks the N-terminal domain that is crucial in MYB30 repression, but carries the C-terminal cysteine protease domain, was investigated. When XopD*_Xcc_*_8004_ (lacking the N-terminal domain) was ectopically expressed in *Arabidopsis* plants, it resulted in plants showing lesion mimic phenotype and exhibiting transcriptional induction of salicylic acid-regulated genes. This finding reveals that impairment in the N-terminal domain of XopD not only eliminates the ability of XopD to suppress host immunity, but it actually converts XopD into an elicitor of host defense responses. It was also discovered that the SUMO protease activity of XopD*_Xcc_*_8004_ was required for these phenotypic responses. Using yeast two-hybrid assay, the *Arabidopsis* transcription factor HFR1 (Long Hypocotyl in Far-Red 1) showed positive interaction with XopD*_Xcc_*_8004_ and the two proteins were colocalized in nuclear bodies of plant cells. Finally, a *HFR1* mutant *Arabidopsis* line exhibited elevated levels of defense-related transcripts and reduced susceptibility to *Xanthomonas campestris*. These results indicate that HFR1 can be a potential nuclear substrate of XopD, modified by its SUMO protease activity and HFR1 represses defense responses in *Arabidopsis* [43]. Biological activity of XopD*_Xcc_*_8004_ has also been linked in *Arabidopsis* to gibberellic acid signaling through the transcriptional regulator DELLA proteins [71]. DELLA proteins are localized in the nucleus and they carry a 17-amino acid-long, highly conserved, N-terminal sequence, the DELLA motif (named after the first five amino acids in this sequence), which plays a crucial regulatory role in sensing the gibberellic acid signal [72,73,74]. Functional aspects of the gibberellin-DELLA interaction and signaling have been summarized here [75]. Currently, *Arabidopsis* DELLA proteins were shown to be nuclear targets of the XopD*_Xcc_*_8004_ cysteine protease domain of XopD type III effector [71]. In this study, XopD*_Xcc_*_8004_ delayed the development of disease symptoms in *P. syringae* pv. *tomato* DC3000-infected *Arabidopsis* leaves, whereas exogenous gibberellic acid treatment was able to reverse this effect to some extent. XopD*_Xcc_*_8004_ also delayed gibberellin-mediated degradation of the RGA DELLA protein, and it was suggested that XopD*_Xcc_*_8004_ might promote plant disease tolerance by partially stabilizing DELLA proteins [71]. DELLA proteins were shown to affect responses of *Arabidopsis* plants to biotrophic and necrotrophic pathogens in an antagonistic manner and they seem to possess integrator roles of salicylic acid and jasmonic acid signaling [76].

Bacterial effector proteins from the HopX1 family (previously AvrPphE) are produced predominantly but not exclusively by various *P. syringae* pathovars [77,78]. When virulence effectors released by a strain of *P. syringae* pv. *tabaci* that does not produce the crucial *P. syringae* toxin coronatine were analyzed, one type III effector, HopX1 of this particular bacterial strain was identified based on its capability to compromise accumulation of the key jasmonic acid pathway repressors the JAZ proteins [44]. The toxin coronatine helps entry of bacteria into the host plant by inducing the opening of stomata [79] and facilitates bacterial growth by inhibiting salicylic acid-related defense pathways through activation of the antagonistic jasmonic acid-dependent pathway [80,81]. It was shown that HopX1 is a cysteine protease and its enzymatic activity is needed for the degradation of JAZ proteins. It was also presented that HopX1 eliminates JAZ proteins in a pathway independent of the jasmonic acid receptor COI1 (Coronatine-insensitive 1) which would also perceive the presence of coronatine. Instead, HopX1 compromises the accumulation of the JAZ family in a specific manner by directly interacting with JAZ proteins through their central ZIM domain in the cytoplasm and nuclei of host cells. Similar to coronatine, HopX1 also activates the jasmonic acid pathway and suppresses salicylic acid-dependent gene expression. By using coronatine deficient pathogen or *COI1* mutant host genotypes, it was presented that HopX1 can complement the deficiency in coronatine production or signaling in order to activate the *Arabidopsis* jasmonate pathway, induce the opening of stomata and promote bacterial pathogenicity. These results suggest that HopX1 contributes to bacterial pathogenicity by mimicking coronatine-induced host cellular responses to trigger plant susceptibility and bacterial HopX1 and coronatine may function redundantly [44].

### 2.6. Protease IV, a Bacterial Lysyl Class Serine Protease Effector

A recent secretome analysis of a *P. aeruginosa* strain (PA14) led to the identification of protease IV, a previously unknown lysyl class serine protease effector and a corresponding novel immune pathway in *Arabidopsis* [45]. Protease IV treatment elicited an immune response comparable to the effect of the MAMP flg22 (a conserved N-terminal epitope of flagellin), characterized by the activation of mitogen-activated protein kinases (MPK3 and MPK6), induction of an oxidative burst, deposition of callose and protection from *P. syringae* pv. *tomato* DC3000 infection. The transcriptomic signature of protease IV treatment was also similar to those gene expression changes that were elicited by flg22. In a search for mechanisms by which protease IV activates an immune response, components of the heterotrimeric G-protein complex were investigated. Indeed, protease IV-triggered immune responses, including the induction of a mitogen-activated protein kinase (MAPK) cascade, were markedly compromised in G-protein mutants, indicating that the G-protein complex may function upstream of a MAPK cascade. Considering further potential signaling components, Receptor for Activated C Kinase 1 (RACK1) emerged as a candidate. The three *Arabidopsis* RACK1 homologs apparently interacted with one subunit (Gβ) of the G-protein complex and also with several members of a MAPK cascade. RACK1 was suggested to function as a scaffold that binds upstream G-protein signaling to downstream activation of a MAPK cascade. Knockdown of *RACK1* genes by using stable RNAi transgenic lines blocked protease IV-mediated defense gene induction and protection from *P. syringae* pv. *tomato* DC3000. Therefore, this novel protease IV-mediated immune pathway is distinct from the previously known flg22 pathway because G-proteins act upstream of a MAPK cascade and the RACK1 protein is uniquely involved here [45].

### 2.7. Alkaline Protease AprA, a Bacterial Zinc Metalloprotease

AprA is an alkaline protease belonging to the serralysin family of zinc metalloproteases. It has been identified in the opportunistic pathogen *P. aeruginosa* and its orthologs have been found in the human pathogen *Serratia marcescens* and the plant pathogens *Dickeya dadantii* or *P. syringae* pv. *tomato* DC3000 [82,83,84,85,86]. In fact, *P. aeruginosa* (and other bacterial species) also express a peptide designated AprI (present in the same operon as AprA) with unclear biological function that acts as an inhibitor of AprA protease [87]. It was shown recently that AprA protease actively degrades monomers of the crucial bacterial MAMP flagellin and the *aprA* operon appears in a highly divergent group of bacterial species [46,88]. An AprA-deficient *P. syringae* pv. *tomato* DC3000 bacterial strain exhibited reduced pathogenicity to *Arabidopsis* when leaves were pressure-infiltrated with the inoculum and this response was dependent on flagellin recognition mediated by the FLS2 receptor. Defense-related transcriptional changes were also markedly higher after inoculation with the AprA-deficient *P. syringae* strain compared to a treatment with the wild type strain. When the bacterial AprA inhibitor AprI was ectopically expressed in *Arabidopsis* the transgenic lines showed reduced susceptibility to wild type *P. syringae* pv. *tomato* DC3000. These results together suggest a sophisticated bacterial virulence mechanism, where AvrA protease is secreted by pathogenic (or even mutualistic) bacteria to eliminate their spilled flagellin molecules by proteolytic degradation and to evade flagellin-mediated apoplastic recognition by the host immune system, helping to establish a beneficial interaction with the host organism [46].

It is worth to mention that *Arabidopsis* cells also encode and synthesize protease inhibitors that inactivate proteases and their roles in plant-pathogen interactions are increasingly understood and appreciated [89,90,91,92,93].

## 3. Conclusions

Classification of protease functions discussed in this work presents a picture where host-derived protease activities can be distinguished as proteolytic functions that contribute to full basal immunity in various *Arabidopsis*-pathogen interactions (CDR1, CathB, CEP1), some that are rather needed for R gene-mediated (effector-triggered) defense (RD19A, PBA1, γVPE), some that regulate systemic immunity and salicylic acid-dependent priming (AED1, SBT3.3) and some that are clearly required for pathogen-elicited PCD (AtMC1, AtMC4, VPEs, CathB, PBA1). XCP2 (in an interaction with PIRIN2) and VPEs were also utilized by *R. solanacearum* or *H. arabidopsidis*, respectively, to establish full microbial pathogenicity in *Arabidopsis*. In these two cases, host-derived proteases contributed to pathogen susceptibility.

Concerning the known functions of pathogen-secreted proteases that are listed here, AvrPphB and AvrRpt2 cysteine protease effectors trigger R gene-mediated immunity in *Arabidopsis* in the presence of their cognate immune receptor R proteins (RPS5 and RPS2). AvrRpt2 by degrading RIN4 and AvrPphB by cleaving PBS1 then perceived by RPS2 or RPS5, respectively, represent effector-target interactions that both exemplify the guard model of effector-receptor recognition. In the absence of corresponding host R proteins, they function as typical virulence effectors. XopD and HopX1 seem to be involved in the suppression of basal immunity in *Arabidopsis*, whereas AprA might participate in bacterial evasion from being perceived by pattern-recognition receptors of the host basal immune system.

It can be also concluded that our knowledge on the cellular targets of pathogen-secreted proteases is rapidly growing, whereas cellular interacting partners of host proteases are still largely elusive [3,94]. The significance of cellular proteolysis and protein metabolism in the regulation of plant biotic stress responses is also emphasized by transcriptomic and proteomic datasets published recently, which show remarkable enrichment of proteolytic factors upon pathogen or salicylic acid treatments [95,96]. The apparent magnitude of *Arabidopsis* or pathogen non-proteasomal proteolytic machineries and their expected functional complexity suggest exciting new future discoveries in the field of regulatory proteases in plant-pathogen interactions.
